# YTHDC1 Modulates the Osteogenic Capacity of hPDLSCs via Wnt/β‐Catenin Signalling Pathway for the Treatment of Bone Defects in Osteoporosis Rats

**DOI:** 10.1111/cpr.70020

**Published:** 2025-03-17

**Authors:** Dan Tan, Qilin Li, Zhenzhen Chen, Hongbing Zhang, Pengcheng Rao, Jingxiang Li, Qianke Tao, Jingang Xiao, Jinlin Song

**Affiliations:** ^1^ Chongqing Key Laboratory of Oral Diseases and Biomedical Sciences, Chongqing Municipal Key Laboratory of Oral Biomedical Engineering of Higher Education College of Stomatology of Chongqing Medical University Chongqing China; ^2^ Luzhou Key Laboratory of Oral & Maxillofacial Reconstruction and Regeneration, the Affiliated Stomatological Hospital Southwest Medical University Luzhou China; ^3^ Department of Periodontal and Mucosal Diseases, the Affiliated Stomatological Hospital Southwest Medical University Luzhou China; ^4^ Department of Oral Implantology, the Affiliated Stomatological Hospital Southwest Medical University Luzhou China

**Keywords:** bone regeneration, hPDLSCs, N6‐methyladenosine, Wnt/β‐catenin signalling pathway, YTHDC1

## Abstract

Human periodontal ligament stem cells (hPDLSCs) have emerged as promising candidates for the treatment of osteoporotic bone defects. Previous studies have indicated that m^6^A plays a crucial role in regulating the osteogenic differentiation of hPDLSCs. However, research on the relationship between YTHDC1, as a reading protein, and the osteogenic differentiation of hPDLSCs remains unexplored. This study aimed to investigate the biological roles of YTHDC1 in the osteogenic differentiation of hPDLSCs and to explore underlying mechanisms. Dot blot analysis revealed a progressive increase in m^6^A methylation during osteogenic differentiation, accompanied by significant upregulation of YTHDC1 expression, as evidenced by qPCR and Western blot. Functional assays utilising siRNA‐mediated knockdown and lentiviral‐mediated overexpression demonstrated that YTHDC1 positively regulated the osteogenic differentiation potential of hPDLSCs. Mechanistically, mRNA‐seq analysis implicated the Wnt/β‐catenin signalling pathway, which was further validated through rescue experiments with the Wnt inhibitor DKK1. Notably, in vivo experiments showed that hPDLSCs overexpressing *YTHDC1* exhibited enhanced bone formation capacity in the osteoporotic rats. In conclusion, our findings suggested that YTHDC1 modulated the osteogenic capacity of hPDLSCs through the Wnt/β‐catenin signalling pathway, highlighting its therapeutic potential for treating bone defects in osteoporotic conditions.

## Graphical abstract

In this study, human periodontal ligament stem cells (hPDLSCs) were successfully isolated and cultivated from the periodontal ligament tissues of healthy premolar teeth. Our in vivo experiments demonstrated that YTHDC1 augmented the osteogenic differentiation capacity of these cells via the Wnt/β‐catenin signalling pathway. Subsequently, hPDLSCs transfected with YTHDC1 and loaded with biphasic calcium phosphate (BCP) were transplanted into bilateral critical‐sized skull defects in osteoporotic (OP) rats to validate their efficacy in vivo. The results indicated that YTHDC1 effectively enhanced the osteogenic differentiation potential of hPDLSCs within the osteogenic microenvironment of OP rats.

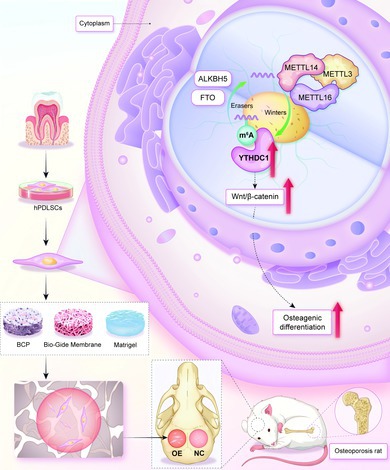

## 1 Introduction

Osteoporosis (OP), a prevalent skeletal disorder characterised by compromised bone strength and heightened vulnerability to fractures, poses a significant health threat across all age groups, yet primarily impacts postmenopausal women and elderly men [1, 2, 3]. The escalating global burden of osteoporotic (OP) fractures, fueled by population ageing, has led to soaring economic costs and a profound societal and familial toll due to increased disability and mortality rates [1, 4, 5]. Current clinical interventions, including drug, physical and exercise therapies, offer symptomatic relief but fall short in effectively restoring pathological fractures and bone defects. Consequently, researchers have increasingly turned their attention to stem cell transplantation as a promising therapeutic avenue for OP [6, 7].

Human periodontal ligament stem cells (hPDLSCs), which are widely used in oral and maxillofacial tissue regeneration due to their easy availability, genetic stability and strong proliferative ability [8, 9, 10, 11, 12], may be an attractive candidate for the treatment of OP. However, the transplantation of these cells into the OP microenvironment confronts challenges, including cellular senescence, which can compromise their proliferation and osteogenic differentiation potential [13]. Enhancing the osteogenic differentiation of seed cells is thus a critical yet elusive goal for clinicians and researchers alike. Recent advancements in gene modification and targeted manipulation have offered promising strategies to bolster the therapeutic efficacy of stem cells [2, 3].

N6‐methyladenosine (m^6^A), a ubiquitous post‐transcriptional RNA modification in eukaryotes, plays a pivotal role in regulating diverse biological processes [14, 15]. Notably, the m^6^A modification has been intimately linked to the pathogenesis of OP [16, 17, 18, 19] and its influence on the osteogenic differentiation of hPDLSCs has garnered attention [20, 21]. Specifically, enzymes involved in m^6^A modification, such as METTL3 and METTL14, have been shown to modulate the osteogenic potential of hPDLSCs [21]. Nevertheless, the regulatory role of YTHDC1, an m^6^A reader protein, remains largely unexplored in this context. YTHDC1, known for its recognition of m^6^A modifications on RNA, has been implicated as a key regulator in various diseases, including cancer and diabetes, through its influence on mRNA degradation, stability, splicing and nuclear export [22, 23, 24, 25]. However, its potential contribution to the osteogenic differentiation of hPDLSCs remains uncharted territory.

The Wnt/β‐catenin signalling pathway stands as a cornerstone in the osteogenic differentiation of stem cells [6, 7, 26]. including hPDLSCs, as evidenced by Wnt3a's stimulatory effects on osteogenic markers and mineralisation [27, 28]. Inhibition of this pathway, either by DKK‐1 or small molecules, leads to diminished osteogenic differentiation, underscoring its critical importance [27]. Furthermore, Wnt/β‐catenin interacts with BMPs and Notch signalling to form a complex regulatory network that modulates the osteogenic response of hPDLSCs [28]. Recent studies have also highlighted oestrogens' role in enhancing hPDLSC osteogenic differentiation, partially through Wnt/β‐catenin activation [29]. However, the intricate interplay between Wnt signalling, YTHDC1 and their precise molecular mechanisms in regulating hPDLSC osteogenic differentiation remains to be fully deciphered.

In this study, we delve into the dynamics of m^6^A modification during osteogenic differentiation of hPDLSCs, observing a notable upregulation of YTHDC1 expression. Employing siRNA‐mediated knockdown and lentivirus‐mediated overexpression (OE), we demonstrate that YTHDC1 positively regulates the osteogenic differentiation of these cells. Through mRNA‐seq and bioinformatics analysis, we uncover a potential mechanism whereby YTHDC1 modulates the osteogenic potential of hPDLSCs via the Wnt/β‐catenin signalling pathway. This finding is further corroborated by a rescue experiment utilising the Wnt signalling inhibitor DKK1. Finally, in vivo experiments utilising an OP rat model reveal that hPDLSCs overexpressing YTHDC1 exhibit enhanced bone formation capabilities in an OP microenvironment. Collectively, our findings underscore the therapeutic potential of YTHDC1 in regulating hPDLSC osteogenic differentiation through the Wnt/β‐catenin signalling pathway, offering a novel strategy for addressing bone defects in OP.

## 2 Materials and Methods

### 2.1 Isolation and Identification of hPDLSCs

This research was approved by the Ethics Committee of Southwest Medical University. hPDLSCs were successfully isolated from periodontal ligament tissues derived from healthy premolars which were specifically chosen from orthodontic patients aged 11–16 years at the Department of Oral and Maxillofacial Surgery, Affiliated Stomatological Hospital of Southwest Medical University. Informed consent forms were signed by all participants. The primary culture of hPDLSCs and the associated experimental procedures were carried out in accordance with the stem cell standards published by Cell Proliferation and the International Society for Stem Cell Research (ISSCR) and its affiliates [30]. Once the third generation of cells reached 70%–80% confluence, they were harvested for flow cytometry analysis using antibodies such as CD31, CD34, CD45, CD73, CD90 and CD105 (Bio‐legend, CA, United States).

### 2.2 Osteogenic Differentiation

hPDLSCs were inoculated in six‐well plates with about 1 × 10^5^ cells per well. After the cells reached the appropriate degree of fusion, osteogenic differentiation was induced by osteogenic induction solution (PD‐014; Pricella). Then the osteogenic induction fluid was renewed every 3 days. After 5 days of induction, the genes and proteins related to osteogenesis were verified by qPCR and WB.

### 2.3 Dot Blot of m^6^A RNA Modification Levels

Total RNA was extracted from cells using Trizol reagent (Ambion), and the RNA concentration in various treatment groups was standardised to the greatest extent possible. The RNA was denatured by incubation at 95°C for 3 min in a PCR apparatus. Subsequently, the denatured samples were promptly transferred onto a nitrocellulose membrane and cross‐linked under a 302 nm UV lamp for 6 min. The membrane was washed with PBS for 5 min, followed by sealing with skim milk. The m^6^A antibody was then incubated at 4°C overnight, and the following day, after washing with PBS, the goat anti‐rabbit IgG‐HRP was incubated at room temperature for 1 h, followed by exposure post‐washing with PBS and ultimately subjected to methylene blue quantitative staining.

### 2.4 siRNA Interference and Lentiviral Transduction

siRNA plasmids and negative control (NC) siRNA were provided by Heyuan Biotechnology Co. Ltd. Before transfection, hPDLSCs were cultured in antibiotic‐free medium. Then siRNA (100 nM) was transfected with Lipofectamine RNAiMAX (Invitrogen) for 6 h, then replaced with fresh growth medium. The silencing efficiency was affirmed by qPCR after 72 h.

Lentivirus for YTHDC1 OE and control vector were constructed by Heyuan Biotechnology Co. Ltd. Lentiviral transfection was performed when the cell fusion reached about 30%. Cell morphologies and fluorescence intensities were observed and recorded using a fluorescence microscope to identify the optimal multiplicity of infection (MOI) following 72 h of lentivirus transduction. Transfection efficiency was subsequently confirmed through qPCR analysis after 72 h.

### 2.5 Alkaline Phosphatase (ALP) and Alizarin Red Staining (ARS)

The third‐generation hPDLSCs were cultured in 12‐well plates and were treated with osteogenic induction solution (PD‐014; Pricella) when the cell fusion reached 80%. ALP activity was detected with the ALP kit (Shanghai Shengong, China) at 3 and 5 days after osteogenesis induction. On Day 15 or 21 of osteogenic induction, calcium nodules were stained with ARS (Cyagen).

### 2.6 RNA Extract and qPCR

RNA was extracted from cells according to the RNA extraction protocol provided by RNAiso Plus (Takara). Then it was reverse transcribed into cDNA by the RevertAid First Strand cDNA Synthesis Kit (Thermo). The sequence of gene primers synthesised by Shenggong Bioengineering Co. Ltd. is shown in Table S1. GAPDH normalisation was used to evaluate the relative expression levels of target genes by 2^−ΔΔ*C*^_t_.

### 2.7 Protein Extract and Western Blot Assay

Total protein extraction of cell samples was performed by the Protein Extraction Kit (Keygen Biotech). These proteins were transferred to polyvinylidene difluoride (PVDF) membranes and incubated with specific antibodies to GAPDH (ab8245), OPN (ab63856), RUNX2 (ab236639), YTHDC1 (ab220159), OSX (ab209484) which were all purchased from abcam, LEF1 (T55350) and β‐CATENIN (T53523) which were purchased from abmart at 4°C overnight. Then, the secondary antibody was incubated at room temperature for 1 h, and then exposed by the ECL system.

### 2.8 mRNA‐Sequencing Analysis

hPDLSCs were cultivated in six‐well plates until they attained the optimal density for lentiviral infection. Subsequently, cell samples were harvested separately from both the OE and NC groups. These cells were lysed using Trizol (supplied by Ambion) and subsequently dispatched to Seqhealth Technology Co. Ltd. in Wuhan, China, for Unique Identifier (UID) RNA‐seq experimentation, high‐throughput sequencing and comprehensive data analysis.

### 2.9 Immunofluorescence Staining

Third‐passage hPDLSCs were cultivated in osteogenic induction medium for a duration of 5 days. Following this, the cells were fixed using paraformaldehyde, subsequently washed with phosphate‐buffered saline (PBS), blocked with skim milk and then incubated overnight at 4°C with both anti‐OPN and anti‐RUNX2 antibodies (both diluted 1:100, sourced from Abcam). On the following day, the cells were incubated with a goat secondary antibody (diluted 1:200, provided by Beyotime) for 1 h. To visualise the cytoskeleton, the cells were stained with phalloidin (diluted 1:100, obtained from Cytoskeleton, USA) for approximately 30 min. The nuclei were then stained with DAPI (sourced from Beyotime, China) for about 15 min. Finally, images were captured using a confocal laser microscope (manufactured by Nikon, Tokyo, Japan).

### 2.10 Cell Proliferation Assessment and Scanning Electron Microscopy Observation

The proliferation of hPDLSCs on the scaffold, following several days of co‐culture, was assessed using the Cell Counting Kit‐8 (CCK‐8, sourced from APExBIO). Subsequently, the dehydrated scaffold samples were subjected to observation under a scanning electron microscope (SEM) to further analyse their morphological characteristics.

### 2.11 Animal Models: Construction of OP Rats

This study was approved by the Ethics Committee of Southwest Medical University and was carried out in strict adherence to the Guidelines for the Care and Use of Experimental Animals issued by the Ministry of Science and Technology of China (2006). A total of 40 female Sprague–Dawley (SD) rats, aged 3–4 weeks, were recruited from Southwest Medical University of Luzhou. The rats were anaesthetised using 1% pentobarbital sodium at a dosage of 30 mg/kg. The OP SD rat model was established through ovariectomy (OVX). After a period of 3 months following OVX, the femurs of both OVX and control rats (Sham Group) were extracted. The femurs were then scanned using micro‐computed tomography (micro‐CT) (SCANCO Medical). Once decalcification was completed, the femur specimens were stained with haematoxylin and eosin (H&E) as well as Masson reagent for further analysis.

### 2.12 Three Different Scaffolds Seeded With hPDLSCs Transplanted Into the Rat Model of Bilateral Critical Size Skull Defect in OP

hPDLSCs were transduced with lentiviruses either overexpressing YTHDC1 or serving as NCs. A 2 mL cell suspension containing approximately 1 × 10^6^ cells was then cultured with three different scaffolds: BCP, Bio‐Gide membrane and Matrigel, in a 12‐well plate for 3 days. Subsequently, the OP rats were divided into four groups based on the scaffold material and osteogenic technique: BCP group, Bio‐Gide membrane group, Matrigel group and Subcutaneous osteogenic group, with 12 rats in each.

Following fixation of the rats in the prone position and disinfection of the designated skin area, bilateral full‐thickness, symmetrical skull defects, each 5 mm in diameter, were carefully drilled. Two groups of hPDLSCs—the OE group and the NC group—were then implanted into the skull defects and subcutaneous skin of the same rat. Specifically, hPDLSCs transduced with YTHDC1‐overexpressing lentiviruses were implanted on one side (e.g., left) of the skull defect, while those transduced with NC viruses were implanted on the opposite side (e.g., right). This paired design allowed for direct comparison between the OE and NC groups, enhancing the scientific rigour of the study.

Twelve weeks post‐implantation, the rats were euthanised and skull and subcutaneous osteogenic specimens were harvested based on the hPDLSCs' transfection status (YTHDC1 OE or control virus). These specimens were then categorised into OE and NC groups for statistical analysis.

### 2.13 Micro‐CT Analysis, H&E Staining and Masson Staining

12 weeks after the transplantation, skull specimens were collected after the rats were euthanised. The skull specimens were scanned by micro‐CT (SCANCO Medical). Then, after decalcification was complete, the specimens were stained by H&E and Masson reagent.

### 2.14 Statistical Analysis

We utilised *t* test or one‐way ANOVA to analyse experimental data; meanwhile, paired *t* test was used to analyse the data of new bone from bilateral skull defects and the representative data were provided with mean ± standard error of the mean and each experiment was repeated at least three times. The analysis and graph preparation were done by Prism GraphPad version 9. When *p* < 0.05 was defined as statistically significant.

## 3 Results

### 3.1 Isolation and Identification of hPDLSCs

**FIGURE 1:**
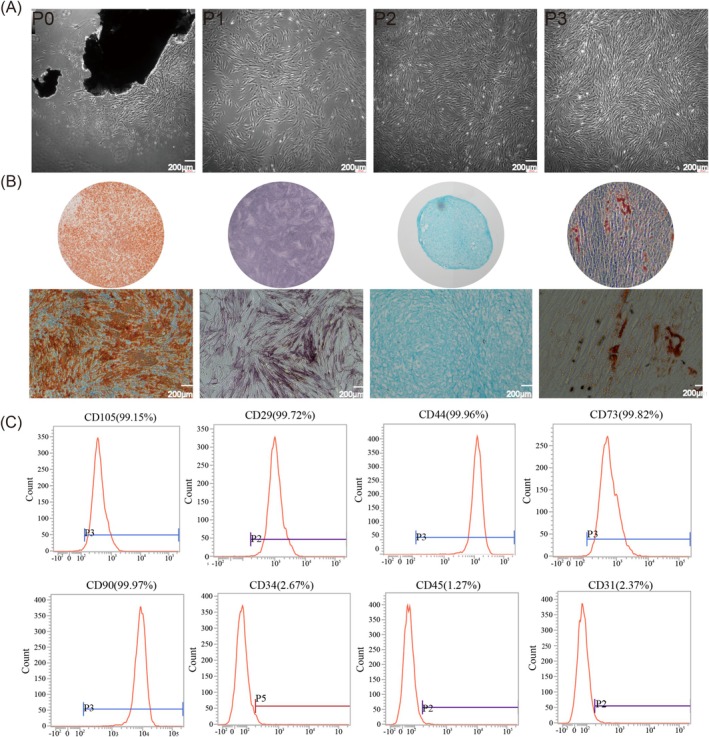
Primary culture and flow cytometry identification of hPDLSCs. (A) Primary culture and subculture of hPDLSCs. The cell morphology was uniformly long and spindle‐like (scale bar =200 μm). (B) Multidirectional differentiation capacity of hPDLSCs: Osteogenic differentiation (ARS and ALP staining), chondrogenic differentiation (Alcian Blue staining) and lipogenic differentiation (Oil red staining) (scale bar =200 μm). (C) Flow cytometry identification of hPDLSCs. The expression of the surface markers: CD73, CD90, CD105, CD31, CD34, CD45 and CD29 (scale bar =200 μm).

hPDLSCs were successfully isolated from periodontal ligament tissues derived from healthy premolars. Microscopic analysis revealed the characteristic long spindle‐shaped morphology of the cells (Figure 1A). The multipotential differentiation capacity of hPDLSCs was verified through induction in multidirectional differentiation media, followed by specific staining (ALP, alizarin red, Alcian blue, oil red O) for osteogenic, adipogenic, chondrogenic and osteogenic lineages (Figure 1B). Flow cytometry confirmed high expression of mesenchymal stem cell (MSC) markers CD73, CD90 and CD105, indicative of stem cell properties such as self‐renewal and multilineage differentiation. Conversely, low expression of haematopoietic stem and progenitor cell (HSPC) markers CD31, CD34 and CD45 ensured high purity by excluding non‐stem cell types, demonstrating the high purity, self‐renewal and differentiation potential of hPDLSCs (Figure 1C).

### 3.2 Upregulation of RNA Methylation and YTHDC1 During Osteoblastic Differentiation of hPDLSCs

**FIGURE 2:**
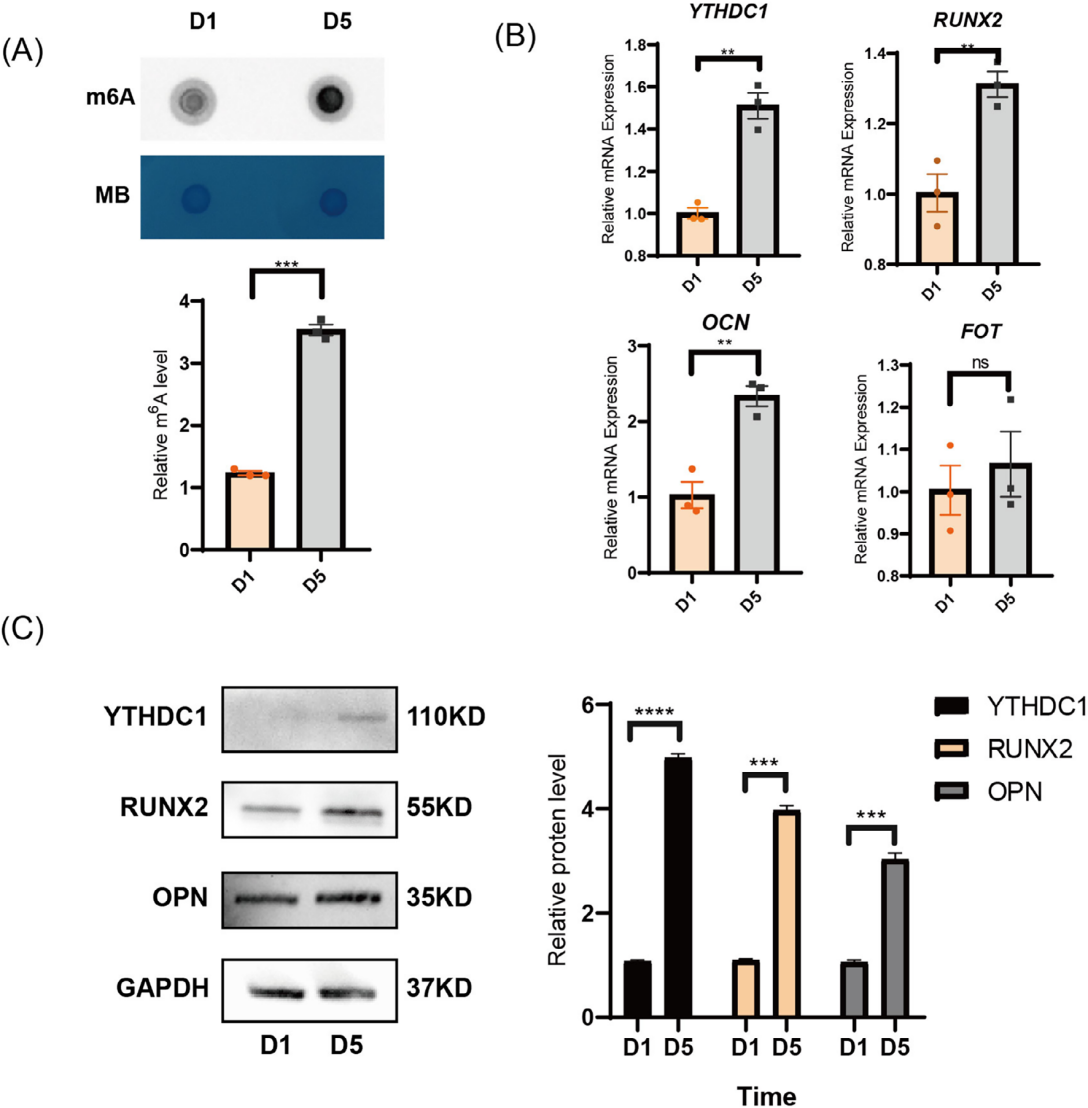
RNA methylation levels and YTHDC1 expression levels were upregulated during osteoblastic differentiation of hPDLSCs. (A) Representative images and quantitative analysis of dot blot of m^6^A on Days 1 and 5 of osteogenic induction. Methylene blue (MB) staining was used as a loading control. (B) qPCR assay was performed to detect the mRNA levels of *YTHDC1, RUNX2* and *OPN* on the first and fifth day of osteogenesis induction. All data are presented as the mean ± SEM. *n* ≥ 3, *p < 0.05, **p < 0.01, ***p < 0.001. (C) The protein levels of YTHDC1, RUNX2 and OPN were detected by WB on the first and fifth day of osteogenesis induction.

To investigate RNA methylation dynamics during osteogenic differentiation, RNA samples were extracted from hPDLSCs at Days 1 and 5 of osteogenic induction. Dot blot analysis revealed significantly deeper and larger hybridisation spots in the Day 5 group, indicating time‐dependent upregulation of m^6^A modification (Figure 2A). qPCR and Western blot experiments confirmed significant changes in m^6^A modification enzymes, particularly the upregulation of YTHDC1, which correlated with the expression of osteogenic markers RUNX2, Osteopontin (OPN) and Osteocalcin (OCN) (Figure 2B,C). These findings suggest a potential role for YTHDC1 in promoting osteogenic differentiation.

### 3.3 *YTHDC1* Regulates the Osteogenic Differentiation of hPDLSCs

**FIGURE 3:**
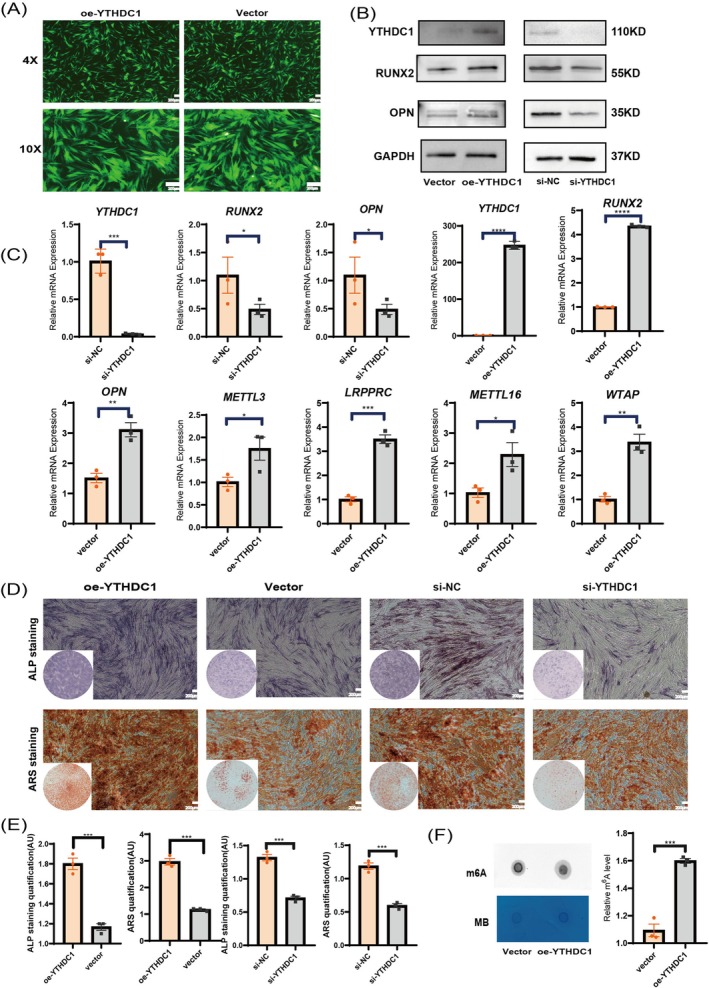
*YTHDC1* regulates the osteogenic differentiation of hPDLSCs. (A) Images captured under a fluorescence microscope revealed the transfection of hPDLSCs with lentiviral vectors overexpressing *YTHDC1* for 3 days (MOI = 40). (B) WB experiments were conducted to detect the protein levels of YTHDC1, as well as osteogenic marker proteins RUNX2 and OPN, in the four groups of cells. (C) qPCR experiments were performed to detect the mRNA levels of *YTHDC1*, osteogenic marker molecules *RUNX2* and *OPN*, as well as some m^6^A modification enzymes in the four groups of cells. (D, E) ALP staining and Alizarin Red staining were performed at 5 and 15 days of osteogenic induction, and subsequent quantitative analysis was carried out using ImageJ. (F) Dot blot assay was used to detect the m^6^A levels in the two groups of cells. All data are presented as the mean ± SEM. *n* ≥ 3, *p < 0.05, **p < 0.01, ***p < 0.001, ****p < 0.0001.

In order to investigate the regulatory role of *YTHDC1* in the osteogenic differentiation of hPDLSCs, Heyuan Biotechnology Co. Ltd. (Shanghai, China) generated si‐RNA mediated knockdown and lentiviral transduction mediated OE of *YTHDC1*. After 72 h, we successfully achieved the knockdown and OE of *YTHDC1*. Cell morphologies and fluorescence intensities were observed and recorded using a fluorescence microscope to identify the optimal MOI (= 40) (Figure 3A). The efficacy of knockdown and OE was confirmed through qPCR and WB experiments. The experimental outcomes distinctly demonstrated that the downregulation of *YTHDC1* resulted in a significant decrease in the expression of the vital osteoblast molecules, *RUNX2* and *OPN*. Conversely, upon OE of YTHDC1, there was a remarkable upregulation of these molecules' expression levels. Furthermore, the enhancement of *YTHDC1* expression triggered notable changes in the mRNA abundance of certain m^6^A modification enzymes (Figure 3B,C). ALP staining and ARS further supported our findings that knocking down *YTHDC1* inhibits osteogenic differentiation potential, while OE promotes osteogenic differentiation potential (Figure 3D,E). Meanwhile, we extracted RNA from cells in the OE group and the vector group, respectively, and conducted dot blot experiments. The results showed that the m^6^A level in the OE group was significantly upregulated compared to the empty vector group (Figure 3F).

### 3.4 mRNA‐Seq Showed That *YTHDC1* Promotes Osteogenic Differentiation Through the Wnt/β‐Catenin Signalling Pathway

**FIGURE 4:**
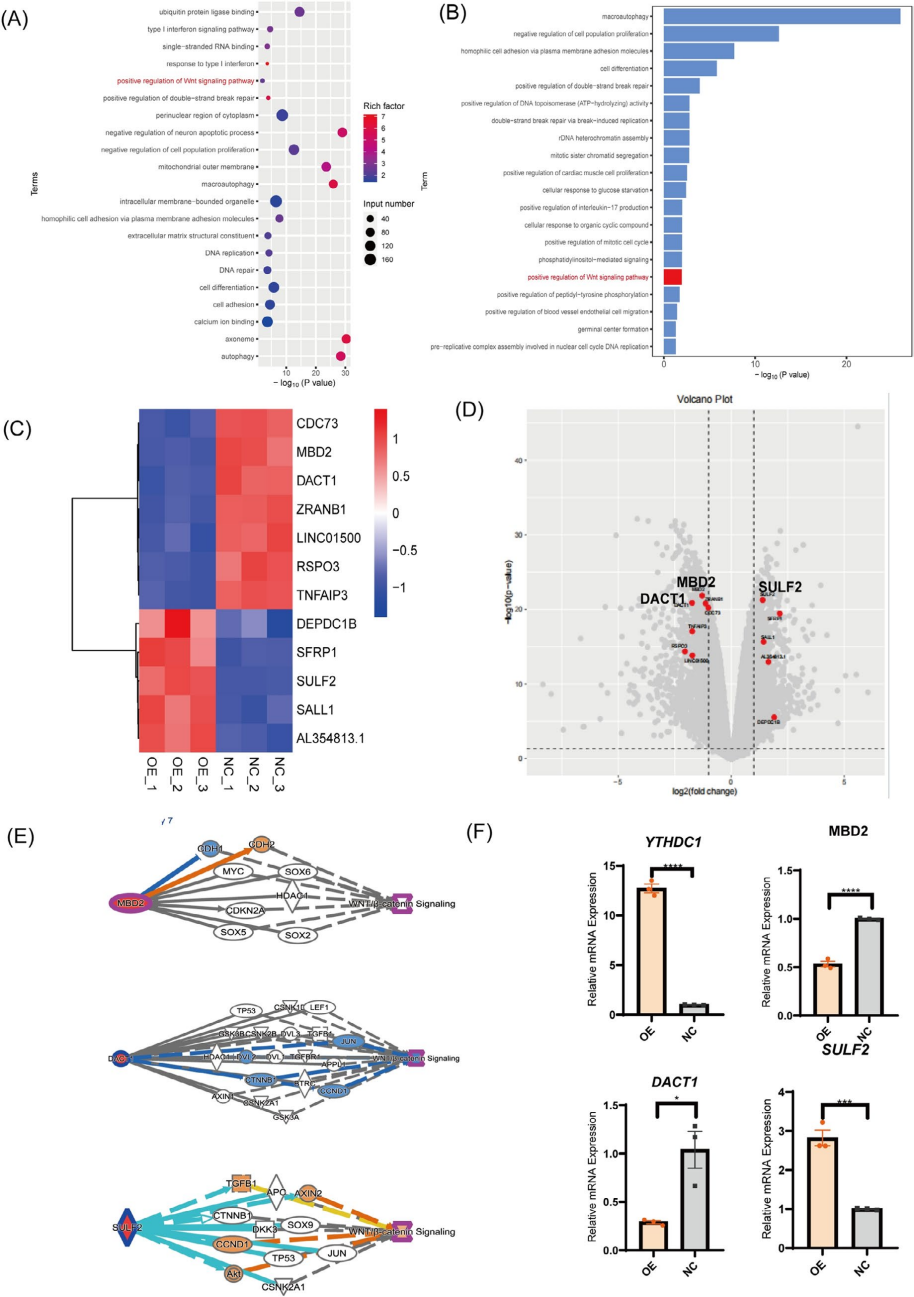
mRNA‐seq showed that YTHDC1 promotes osteogenic differentiation through the Wnt/β‐catenin signalling pathway, a finding corroborated by both IPA and qPCR validation. (A, B) KEGG signalling pathway analysis identified the Wnt/β‐catenin signalling pathway as significantly enriched in two groups. (C, D) Heatmap and volcano plot analysis revealed significant up‐ or downregulation of 12 target genes within the Wnt signalling pathway, including *MDB2, DACT1* and *SULF2*. (E) IPA analysis confirmed the activation or inhibition of the Wnt signalling pathway by three of the target genes (*MDB2, DACT1* and *SULF2*) through multiple pathways. (F) The results of mRNA‐seq were verified by qPCR. All data are presented as the mean ± SEM. *n* ≥ 3, *p < 0.05, **p < 0.01, ***p < 0.001, ****p < 0.0001.

Previous research has established the pivotal role of *YTHDC1* in promoting osteogenic differentiation in hPDLSCs. Nevertheless, its underlying mechanism of action was yet to be fully elucidated. Subsequently, we performed mRNA‐seq analysis on overexpressed groups (OE groups) and NC groups to elucidate the underlying mechanism. Upon bioinformatic analysis, KEGG signalling pathway assessment revealed an enrichment of differentially expressed genes specifically within the Wnt/β‐catenin signalling pathway (Figure 4A,B). The meticulous depiction of these findings through a heatmap and volcano plot unveiled disparities in the expression profiles of 12 pivotal target genes residing within the Wnt/β‐catenin signalling pathway, comparing the two distinct cellular groups (Figure 4C,D). To substantiate the sequencing outcomes, we subsequently embarked on ingenuity pathway analysis (IPA) and complemented it with qPCR experiments, ensuring the robustness and reliability of our findings (Figure 4E,F).

### 3.5 *YTHDC1* ‐OE Upregulates the Osteogenic Ability of PDLSCs via Wnt/β‐Catenin Signalling Pathway In Vitro

**FIGURE 5:**
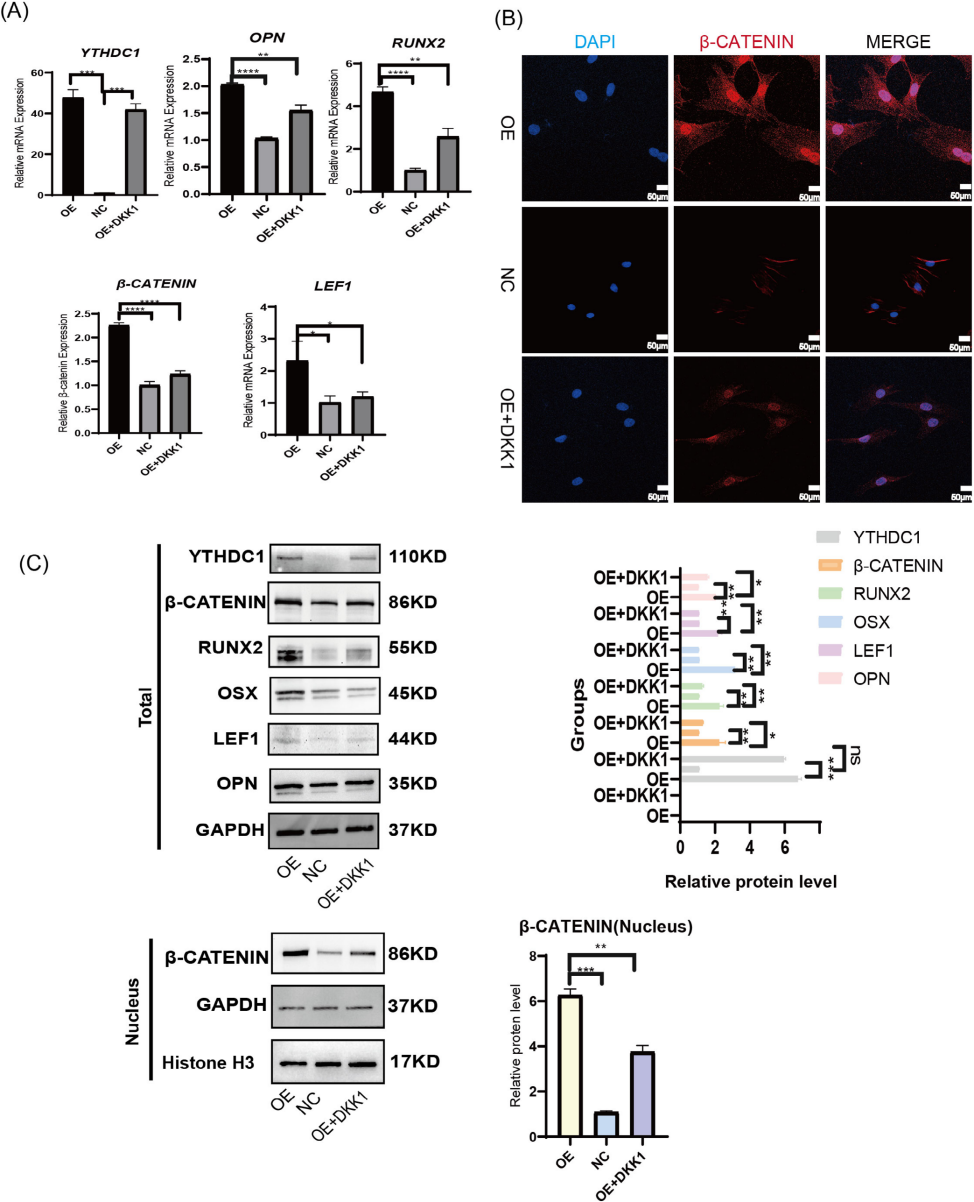
YTHDC1 overexpression activates the Wnt/β‐catenin signalling pathway. After 5 days of osteogenic induction for the three groups of cells, a qPCR experiment was performed to analyse the mRNA expression levels of *YTHDC1*, osteogenic marker molecules and Wnt/β‐catenin signalling pathway molecules. (B) Immunofluorescence staining for β‐catenin after osteogenic induction for 5 days. (C) After 5 days of osteogenic induction for the three groups of cells, a WB experiment was conducted to analyse the expression levels of YTHDC1, osteogenic marker proteins and Wnt/β‐catenin signalling pathway proteins.

**FIGURE 6:**
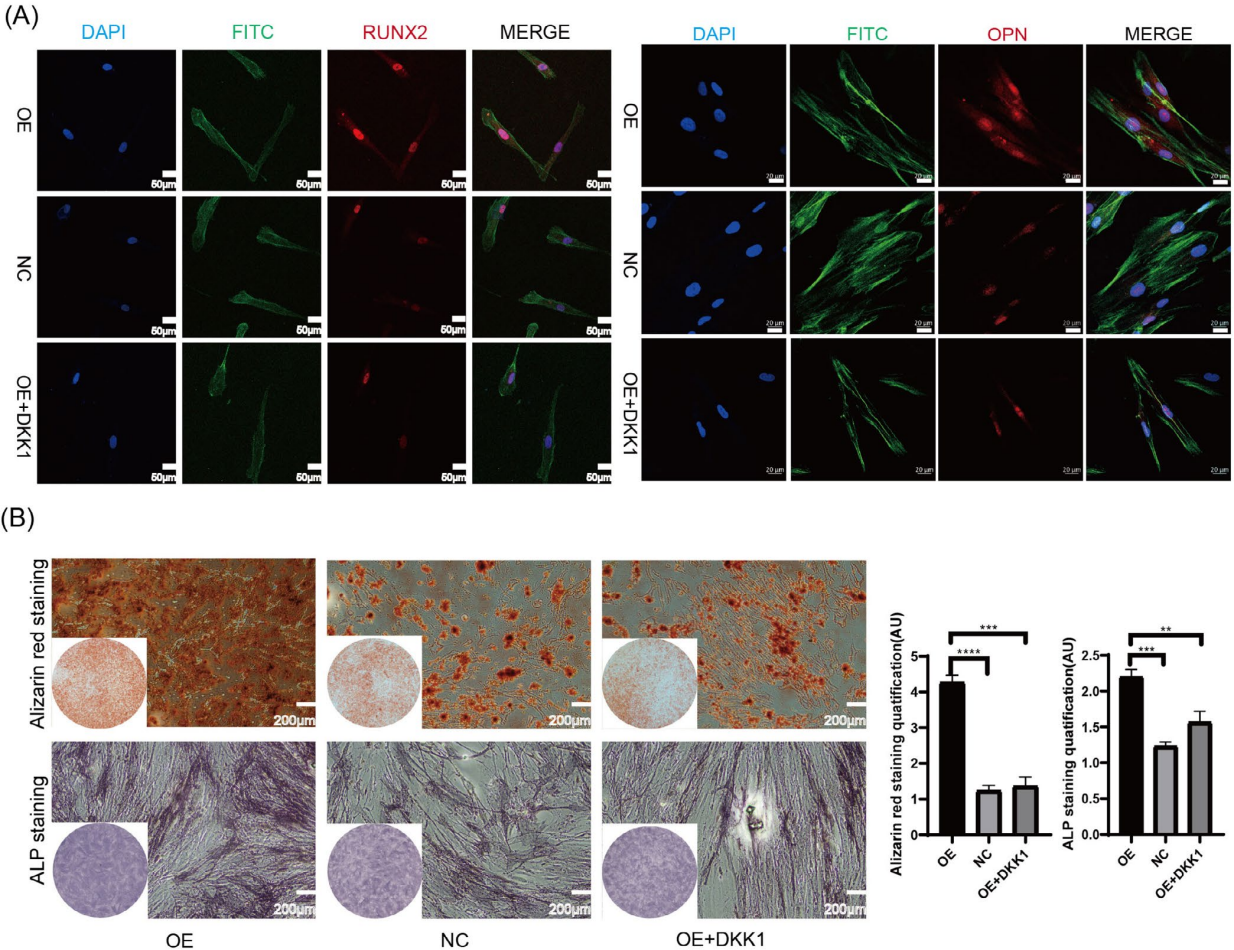
YTHDC1 overexpression upregulates the osteogenic ability of PDLSCs via the Wnt/β‐catenin signalling pathway. (A) Immunofluorescence staining for RUNX2 and OPN after osteogenic induction for 5 days. (B) Alizarin red staining and ALP staining showed the osteogenic differentiation ability of three groups after osteogenic induction for 15 and 5 days. Semi‐quantitative analysis of them by utilising ImageJ. All data are presented as the mean ± SEM. *n* ≥ 3, *p < 0.05, **p < 0.01, ***p < 0.001, ****p < 0.0001.

In addition, we used Dickkopf WNT Signalling Pathway Inhibitor 1 (DKK1) to detect whether YTHDC1 promotes osteogenic differentiation through the Wnt/β‐catenin signalling pathway. The cells were categorised into three groups: the OE group (overexpressing *YTHDC1*), the NC group and the OE + DKK1 group (*YTHDC1* OE followed by DKK1 treatment). Combined with references [7] and previous experiments, we used CCK8 reagents to screen DKK1 concentration and finally determined that a 100 nM concentration was the optimal drug administration concentration. First, the qPCR results indicated that OE of *YTHDC1* activated the Wnt signalling pathway and significantly upregulated osteogenic marker molecules compared to the control group. Upon addition of DKK1, despite no significant change in *YTHDC1* expression, the Wnt signalling pathway was markedly inhibited, leading to a significant downregulation of osteogenic markers such as *RUNX2* and *OPN* (Figure 5A). Immunofluorescence staining for β‐catenin, conducted following 5 days of osteogenic induction, revealed a notably higher expression in the OE group relative to the other two groups. Specifically, β‐catenin in the OE group exhibited a pronounced nuclear heterotopia phenomenon (Figure 5B). To further validate these observations, WB analysis was performed, which corroborated the qPCR findings and was in line with the cellular immunofluorescence results. This analysis demonstrated a statistically significant upregulation of β‐catenin expression in the nuclear proteins of the OE group compared to the other two groups (Figure 5C). Additionally, immunofluorescence staining for RUNX2 and OPN after 5 days of osteogenic induction indicated a higher expression level in the OE group compared to the control groups (Figure 6A). Finally, ALP and ARS assessments revealed that the OE group exhibited stronger osteogenic capabilities, as evidenced by deeper staining, more calcium nodules and enhanced bone formation compared to the NC group. However, the osteogenic capability of the OE + DKK1 group was significantly reduced compared to the OE group after the addition of DKK1 (Figure 6B). In summary, the methylase YTHDC1 plays a role in promoting osteogenic differentiation via the Wnt/β‐catenin signalling pathway.

### 3.6 YTHDC1 Can Promote Bone Repair in OP Rats In Vivo

To establish the OP SD rat model, OVX was performed. Three months post‐OVX surgery, the femurs of both OVX rats and the control group underwent micro‐CT scanning (SCANCO Medical) (Figure S1A). Notably, in the OP group, there was a pronounced decrease in bone volume fraction (BV/TV), trabecular number (Tb.N) and trabecular bone mineral density (Tb.BMD). Conversely, trabecular separation (Tb.Sp) showed an upward trend (Figure S1B). Following decalcification, the femur specimens were stained with H&E and Masson reagent (Figure S1C), confirming the successful establishment of the OP SD rat model. Subsequently, the OP rats were positioned prone, their skin was disinfected, and bilateral skull holes were drilled to create two full‐thickness, symmetrical skull defects with a diameter of 5 mm (Figure S1D).

In vivo, we implanted three different scaffolds, containing hPDLSCs overexpressing *YTHDC1* and vector lentivirus, into the two 5 mm full‐thickness symmetrical skull defects of the OP rats.

Initially, the CCK‐8 assay confirmed the growth and proliferation of hPDLSCs on the three distinct scaffolds (Figure S2A). After 3 days of co‐culture, DAPI staining and confocal microscopy verified the seeding of hPDLSCs on BCP (Figure S2B). Simultaneously, SEM was employed to confirm the effective adhesion between hPDLSCs and the scaffold (Figure S2C), facilitating the transplantation of seed cells. Ultimately, hPDLSC‐seeded scaffolds were transplanted onto the bilateral critical‐sized calvarial defects of OP rats. Samples were collected 12 weeks post‐transplantation for further analysis. To further validate the regulatory effect of YTHDC1 on ectopic bone formation in OP rats, we attempted subcutaneous ectopic bone formation by co‐culturing hPDLSCs overexpressing YTHDC1 and those with empty vectors, respectively, with BCP material for 3 days. These were then implanted into the subcutaneous tissue of the backs of OP rats.

**FIGURE 7:**
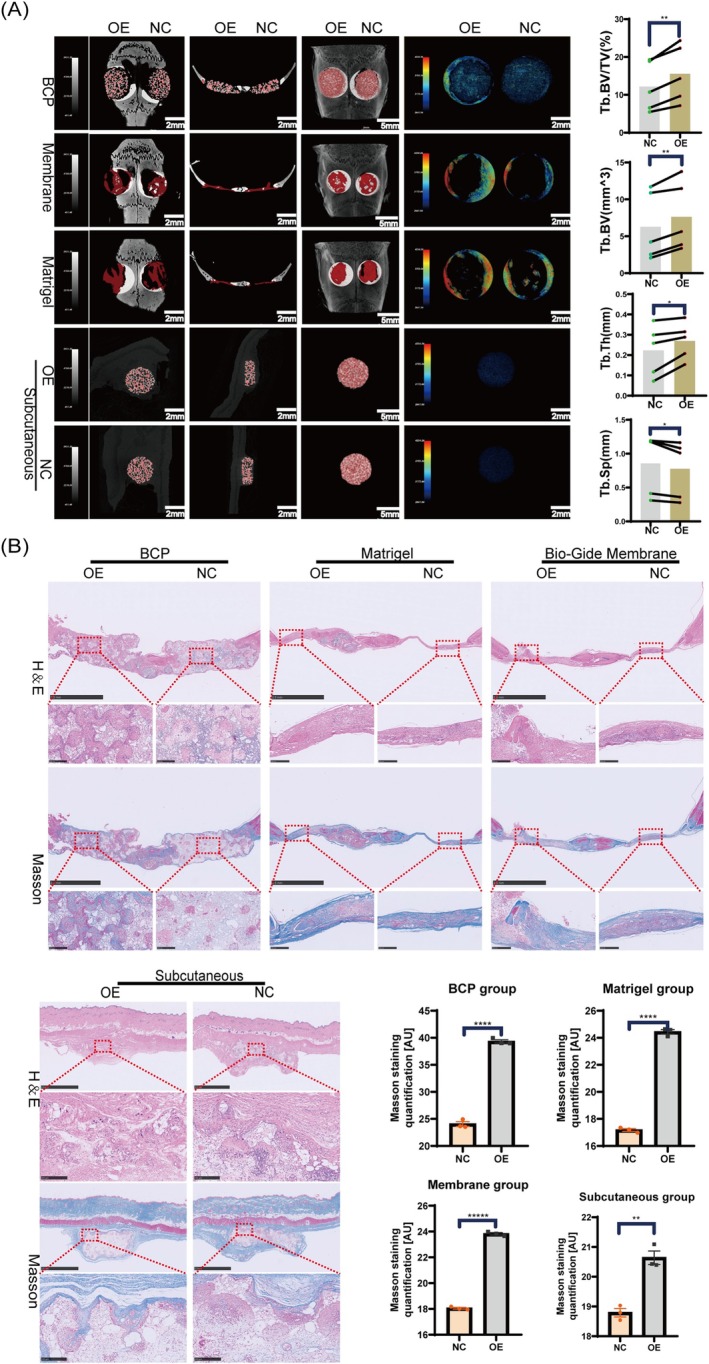
Overexpression of *YTHDC1* promotes osteogenic differentiation of hPDLSCs. (A) At 12 weeks after transplantation of hPDLSCs supported by three different scaffolds, micro‐CT showed representative images of bilateral skull defect bone repair and subcutaneous ectopic bone formation in OP rats. The bone parameters (BV/TV, BV, Th) of the two groups were analysed statistically and there were significant differences. (B) Representative images of H&E and Masson staining at 12 weeks after hPDLSCs transplantation with different scaffolds in 2 groups. Semi‐quantitative analysis of H&E and Masson staining using ImageJ. All data are presented as the mean ± SEM. *n* ≥ 3, *p < 0.05, **p < 0.01, ***p < 0.001, ****p < 0.0001.

Micro‐CT analysis, conducted at 12 weeks post‐transplantation, revealed a significant increase in bone matrix levels in the OE group. Specifically, micro‐CT demonstrated that bone analysis parameters such as BV/TV, BV and Th increased significantly, while SP decreased in the OE group after 12 weeks of hPDLSCs transplantation. These differences were statistically significant (Figure 7A). Furthermore, H&E and Masson staining showed that, at 12 weeks post‐transplantation, the OE group exhibited significantly more fibrosis and mineralised new bone compared to the NC group. Semi‐quantitative analysis of H&E and Masson staining using ImageJ confirmed that these differences were statistically significant (Figure 7B). In conclusion, the OE of *YTHDC1* enhances the osteogenic capacity of hPDLSCs.

**FIGURE 8:**
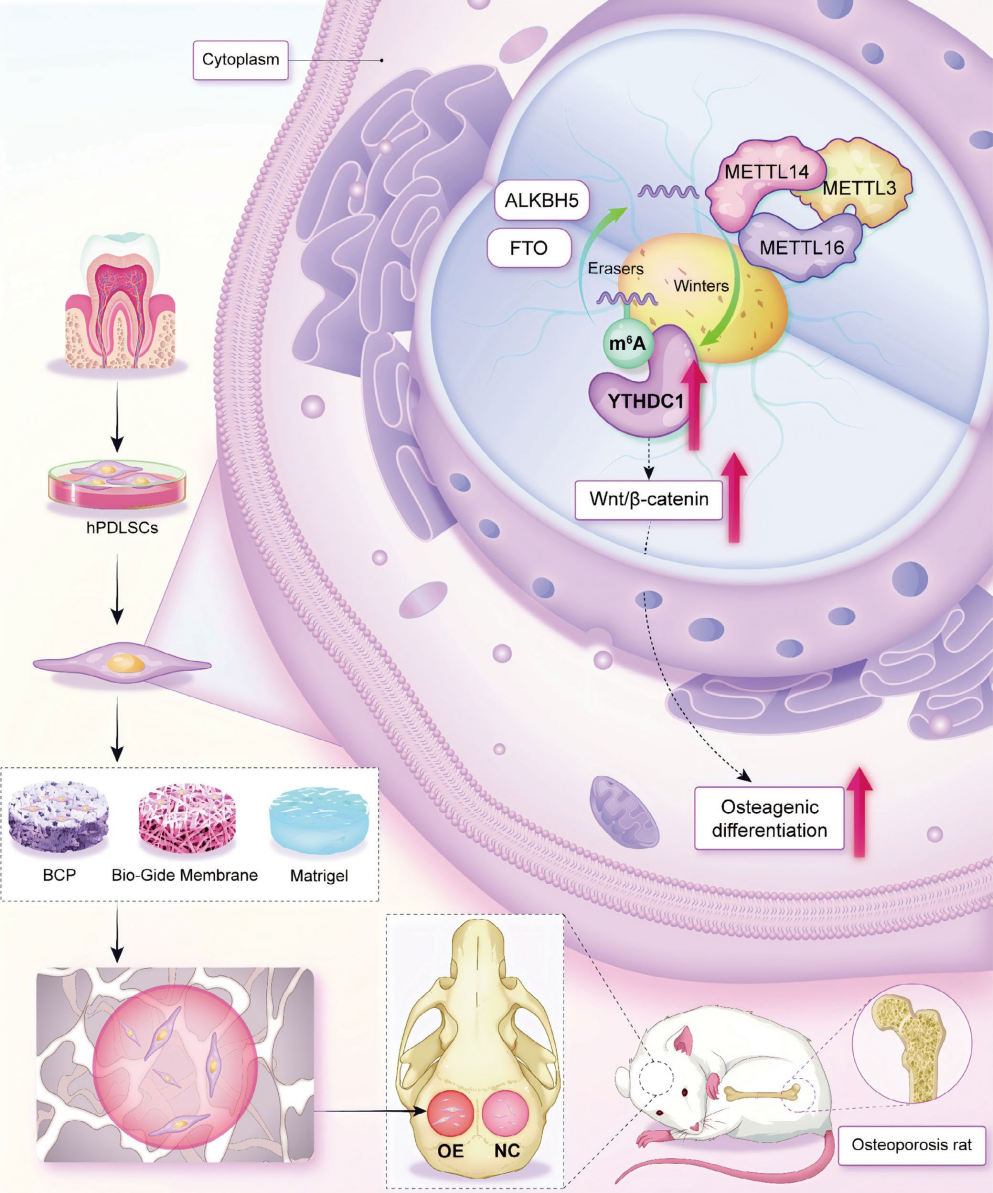
Schematic illustration of YTHDC1 regulating the osteogenic differentiation ability of hPDLSCs via the Wnt/β‐catenin signalling pathway for the treatment of bone defects in osteoporotic rats.

In summary, the osteogenic differentiation of hPDLSCs is modulated by the epigenetic modification m^6^A, exhibiting a time‐dependent pattern. Notably, the expression of YTHDC1, an intranuclear reading protein, is markedly elevated during this process. We hypothesised that YTHDC1 plays a pivotal role in regulating the osteogenic differentiation of hPDLSCs. To validate this, we conducted both knockdown and OE experiments, which confirmed that *YTHDC1* indeed promotes osteogenic differentiation in hPDLSCs. Furthermore, using a combination of high‐throughput sequencing and low‐throughput verification, we elucidated that YTHDC1 exerts its effect through the Wnt/β‐catenin signalling pathway (Figure 8). Finally, we extended our findings to an in vivo model of bilateral skull defects in OP rats, demonstrating that YTHDC1 retains its ability to enhance osteogenic differentiation of hPDLSCs within the OP microenvironment, thus offering a potential therapeutic approach for treating OP bone defects in rats.

## 4 Discussion

OP is a systemic condition that frequently impacts postmenopausal women and the elderly, characterised by heightened bone turnover, compromised bone microstructure, reduced bone mass and bone mineral density [31]. The ageing population has led to a growing concern regarding OP as a significant public health issue [1, 6, 7]. Recent research has shown promising results in utilising stem cell transplantation for the treatment of OP, with stem cells demonstrating the ability to address the condition through mechanisms such as self‐osteogenic differentiation, anti‐inflammatory properties and paracrine effects [32, 33, 34, 35, 36, 37]. In the field of stomatology, researchers have identified hPDLSCs as a promising source of seed cells due to their accessibility, genomic stability and robust proliferation [8, 9, 10, 11, 12]. Previous studies have supported the potential of these cells, leading to the establishment of a cell bank for regenerative medicine and a growing focus on cell therapy in current research efforts [38, 39, 40, 41]. Transplantation into an OP microenvironment induces cellular senescence in seed cells, subsequently impacting their proliferation and differentiation [13], findings which align with prior research. We also encountered the same dilemma in our previous experiments. Therefore, enhancing the osteogenic differentiation ability of seed cells through genetic modification to promote the therapeutic effect of bone defect areas after seed implantation is a valuable research topic.

At the inception of our study, dot blot hybridization experiments demonstrated a progressive increase in methylation during the osteogenic differentiation of hPDLSCs. This observation aligns with previous findings that established the intricate regulation of hPDLSCs osteogenic differentiation by m^6^A modification. Specifically, the m^6^A modifying enzymes, METTL3 and METTL14, have been identified as pivotal regulators of the osteogenic differentiation potential of hPDLSCs [21]. PCR and WB experiments showed that YTHDC1 was significantly upregulated during osteogenic differentiation. Therefore, we hypothesised that the increased bone differentiation of hPDLSCs was related to the upregulation of YTHDC1. To validate our hypothesis, we conducted knockdown and OE experiments targeting *YTHDC1*. The results consistently showed that YTHDC1 modulates the osteogenic differentiation potential of hPDLSCs, corroborating findings from a study published in Nature by Chen and colleagues (2021), which first revealed that the absence of YTHDC1 prompts mouse embryonic stem cells (mESCs) to revert to a state resembling the two‐cell stage, highlighting the essentiality of YTHDC1 in maintaining mESC identity [15]. Furthermore, the functionality of YTHDC1 is contingent upon its recognition of m^6^A modifications, reinforcing the intricate interplay between YTHDC1, m^6^A modification and stem cell differentiation [22, 23, 24, 25]. Meanwhile, an article entitled ‘Nuclear m^6^A reader YTHDC1 promotes muscle stem cell activation/proliferation by regulating mRNA splicing and nuclear output’ published in eLife in 2023 further supports the idea that YTHDC1 promotes muscle stem cell activation and proliferation by participating in the regulation of nuclear output and mRNA splicing [42]. Drawing upon our research findings, we have, for the first time, elucidated the pivotal regulatory function of YTHDC1 in the osteogenic differentiation of hPDLSCs. This discovery aligns seamlessly with analogous research outcomes pertaining to other types of stem cells, reinforcing the significance and universality of our findings.

The crucial role of the Wnt/β‐catenin pathway in osteogenic differentiation of MSCs has been amply confirmed [26, 43]. Numerous previous studies have also shown that the osteogenic differentiation of hPDLSCs is similarly regulated by the Wnt signalling pathway [27, 28]. Moreover, the crosstalk between Wnt signalling and other osteogenic regulatory networks, such as BMP, Notch and TGF‐β pathways, further underscores the complexity and intricacy of this process [27, 28, 29, 44]. In summary, the existing literature strongly supports the notion that the Wnt/β‐catenin pathway plays a critical role in regulating the osteogenic differentiation of hPDLSCs. In this study, in order to elucidate the underlying mechanism of YTHDC1 in regulating osteogenic differentiation of hPDLSCs, we conducted mRNA‐seq. Upon bioinformatic analysis, KEGG signalling pathway assessment revealed an enrichment of differentially expressed genes specifically within the Wnt/β‐catenin signalling pathway and verified it through qPCR experiments, WB experiments and cellular immunofluorescence experiments, confirming the regulatory role of the Wnt/β‐catenin signalling pathway in osteogenic differentiation of hPDLSCs, which is consistent with previous research findings. Based on our research results, we have identified for the first time that YTHDC1 regulates osteogenic differentiation of hPDLSCs through the Wnt signalling pathway. Subsequently, using OP rats, we established models of bilateral cranial defects and subcutaneous ectopic bone formation to validate that YTHDC1 remains capable of promoting bone formation by hPDLSCs within the OP microenvironment. These findings were consistent with those of in vitro experiments.

In conclusion, we confirmed that YTHDC1 regulates osteogenic differentiation of hPDLSCs from multiple dimensions such as knockdown and OE. Meanwhile, RNA‐seq and DKK1 design rescue experiments confirmed that YTHDC1 regulates osteogenic differentiation through the Wnt/β‐catenin signalling pathway. Finally, through OP rats, we verified that YTHDC1 remains capable of promoting bone formation by hPDLSCs within the OP microenvironment. As widely recognised, YTHDC1, as a reader, functions by recognising m^6^A modifications on mRNAs or non‐coding RNAs, subsequently mediating diverse downstream events through mechanisms such as modulating alternative splicing, regulating mRNA degradation, stabilising mRNAs and controlling the nuclear export of these molecules. Consequently, delving into the identification of YTHDC1's target molecules and validating the specific pathways through which it exerts its effects constitutes the primary focus of our subsequent research endeavours.

## Author Contributions

D.T. designed the experiments, performed the experiments, analysed the data and prepared the manuscript, drafted the manuscript and wrote the manuscript. Q.L. performed the collection of data, the analysis of data, reviewed and revised the manuscript. Z.C. performed data analysis and interpretation. H.Z. contributed to helping conduct animal experiments. P.R., J.L. and Q.T. contributed to data collection and interpretation. J.X. and J.S. directed the project, provided financial support, critically revised the manuscript and gave final approval.

## Ethics Statement

The animal experiments performed were approved by the Laboratory Animal Center of Southwest Medical University (20220701–004). The acquisition of hPDLSCs used in the experiment was approved by the Ethics Committee of the Affiliated Stomatological Hospital of Southwest Medical University (20220701–002).

## Conflicts of Interest

The authors declare no conflicts of interest.

## Supporting information


**Figure S1.** Establishment of bilateral critical‐sized calvarial defects in OP rats. (A, B) Micro‐CT imaging demonstrated osteoporosis in the femurs of rats in the OVX group, accompanied by significant downregulation of bone analysis parameters. (C) The results of HE staining and Masson staining of rat femurs in the OP group and CON group, along with their semi‐quantitative analysis. (D) Bilateral critical size skull defect was established in OP rats with gingival circumferential knife. All data are presented as the mean ± SEM. *n* ≥ 3, *p < 0.05, **p < 0.01, ***p < 0.001. ****p < 0.000 1.

**Figure S2.** hPDLSCs could proliferate and seed on the three scaffolds. (A) CCK‐8 reagent detected the proliferation of hPDLSCs on the three scaffolds at different times. (B) DAPI staining for hPDLSCs on BCP by Confocal microscope. (C) The effective adhesion of hPDLSCs to three scaffolds was verified by SEM.

**Table S1.** Primer Sequences for qPCR.

## Data Availability

The data that support the findings of this study are available from the corresponding author upon reasonable request.
