# Supplementary figures and images for: YTHDC1 Modulates the Osteogenic Capacity of hPDLSCs via Wnt/β‐Catenin Signalling Pathway for the Treatment of Bone Defects in Osteoporosis Rats
Source: Cell Prolif. 2025 Mar 17;58(8):e70020. doi: 10.1111/cpr.70020 (PMC12336452; doi:10.1111/cpr.70020)

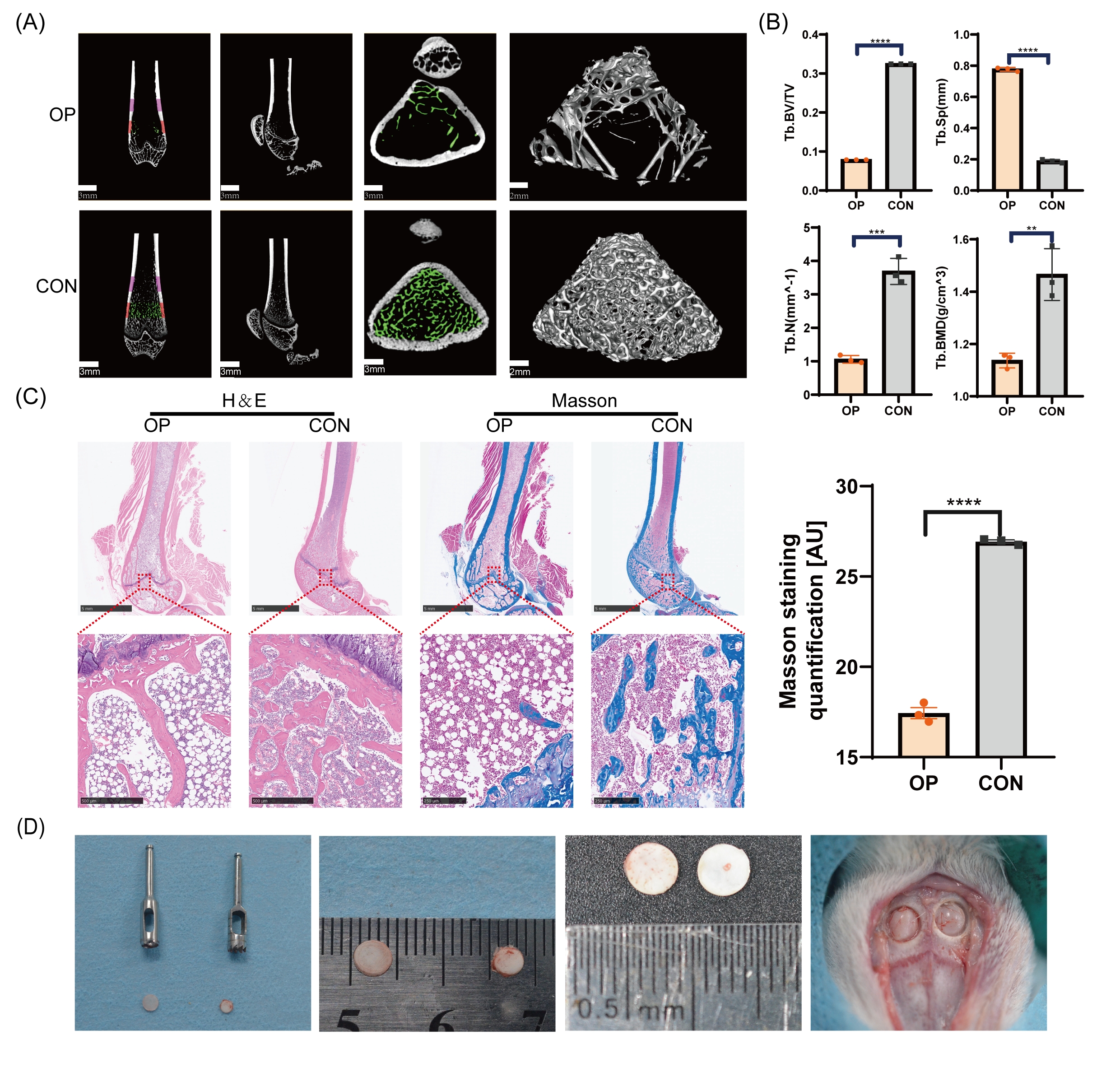

Supplement: Supplementary file 1 — Figure S1. Establishment of bilateral critical‐sized calvarial defects in OP rats. (A, B) Micro‐CT imaging demonstrated osteoporosis in the femurs of rats in the OVX group, accompanied by significant downregulation of bone analysis parameters. (C) The results of HE staining and Masson staining of rat femurs in the OP group and CON group, along with their semi‐quantitative analysis. (D) Bilateral critical size skull defect was established in OP rats with gingival circumferential knife. All data are presented as the mean ± SEM. n ≥ 3, *p < 0.05, **p < 0.01, ***p < 0.001. ****p < 0.000 1. [file CPR-58-e70020-s002.jpg]

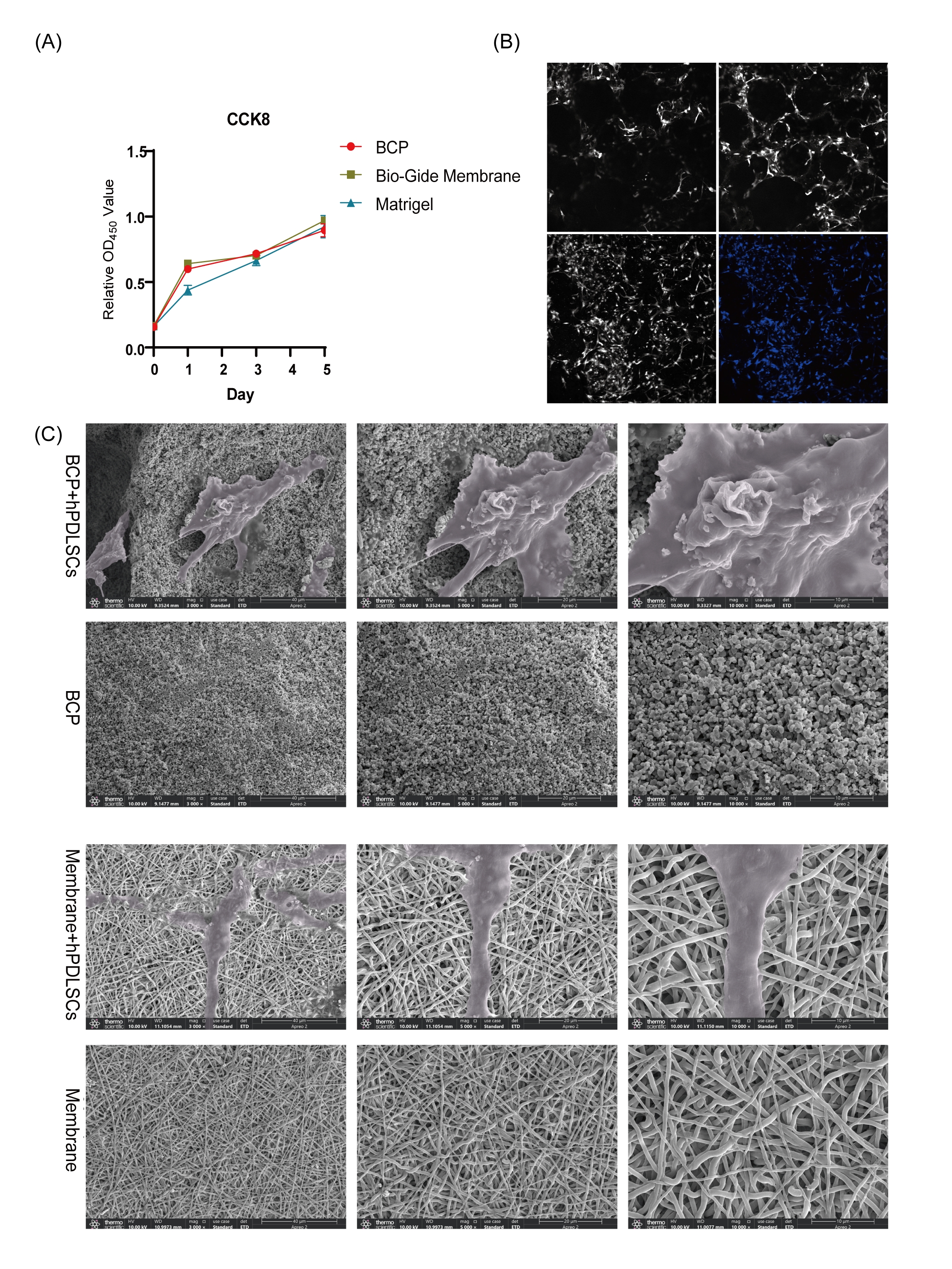

Supplement: Supplementary file 2 — Figure S2. hPDLSCs could proliferate and seed on the three scaffolds. (A) CCK‐8 reagent detected the proliferation of hPDLSCs on the three scaffolds at different times. (B) DAPI staining for hPDLSCs on BCP by Confocal microscope. (C) The effective adhesion of hPDLSCs to three scaffolds was verified by SEM. [file CPR-58-e70020-s001.jpg]
