# Supplementary material for: YTHDC1 Modulates the Osteogenic Capacity of hPDLSCs via Wnt/β‐Catenin Signalling Pathway for the Treatment of Bone Defects in Osteoporosis Rats
Source: Cell Prolif. 2025 Mar 17;58(8):e70020. doi: 10.1111/cpr.70020 (PMC12336452; doi:10.1111/cpr.70020)
Supplement: Supplementary file 3 — Table S1. Primer Sequences for qPCR. [file CPR-58-e70020-s003.docx]

**Table S1 Primer Sequences for qPCR**

| **Genes** | **Primer sequence 3’-5’** |
| --- | --- |
| *GAPDH* | F:GGAGCGAGATCCCTCCAAAAT |
|  | R:GGCTGTTGTCATACTTCTCATGG |
| *RUNX2* | F:TGGTTACTGTCATGGCGGGTA |
|  | R:TCTCAGATCGTTGAACCTTGCTA |
| *YTHDC1* | F:AACTGGTTTCTAAGCCACTGAGC |
|  | R:GGAGGCACTACTTGATAGACGA |
| *OPN* | F:CTCCATTGACTCGAACGACTC |
|  | R:CAGGTCTGCGAAACTTCTTAGAT |
| *METTL3* | F:TTGTCTCCAACCTTCCGTAGT |
|  | R:CCAGATCAGAGAGGTGGTGTAG |
| *OCN* | F: CACTCCTCGCCCTATTGGC |
|  | R:CCCTCCTGCTTGGACACAAAG |
| *METTL16* | F:CTCTGACGTGTACTCTCCTAAGG |
|  | R:TACCAGCCATTCAAGGTTGCT |
| *WTAP* | F:CTTCCCAAGAAGGTTCGATTGA |
|  | R:TCAGACTCTCTTAGGCCAGTTAC |
| *FOT* | F:ACTTGGCTCCCTTATCTGACC |
|  | R:TGTGCAGTGTGAGAAAGGCTT |
| *LRPPRC* | F:CGGAGGACTACTGAGCCCA |
|  | R:AGCGGCAGGTATCATTAAAAACT |
| *HNRNPA2B1* | F:ATTGATGGGAGAGTAGTTGAGCC |
|  | R:AATTCCGCCAACAAACAGCTT |
| *ZC3H13* | F:TCTGATAGCACATCCCGAAGA |
|  | R:CAGCCAGTTACGGCACTGT |
| *β-CATENIN* | F:CCTATGCAGGGGTGGTCAAC |
|  | R:CGACCTGGAAAACGCCATCA |
| *LEF1* | F:AGAACACCCCGATGACGGA |
|  | R:GGCATCATTATGTACCCGGAAT |
| siRNA | F:GAAGCAAGAUACAGAGAUATT |
|  | R:UAUCUCUGUAUCUUGCUUCTT |
| siRNA-NC | F:UUCUCCGAACGUGUCACGUTT |
|  | R:ACGUGACACGUUCGGAGAATT |
